# Differential regulation of the Wnt/β-catenin pathway by hepatitis C virus recombinants expressing core from various genotypes

**DOI:** 10.1038/s41598-018-29078-2

**Published:** 2018-07-25

**Authors:** Stephanie Aicher, Athanasios Kakkanas, Lisette Cohen, Brigitte Blumen, Gabriela Oprisan, Richard Njouom, Eliane F. Meurs, Penelope Mavromara, Annette Martin

**Affiliations:** 1 Institut Pasteur, Unit of Molecular Genetics of RNA Viruses, Paris, France; 2 CNRS UMR3569, Paris, France; 3 Université Paris Diderot-Sorbonne Paris Cité, Paris, France; 4 Hellenic Pasteur Institute, Athens, Greece; 5 University of Patras, School of Health Sciences, Department of Pharmacy, Patras, Greece; 6 Cantacuzino National Medical-Military Institute of Research and Development, Molecular Epidemiology Laboratory, Bucharest, Romania; 7 Titu Maiorescu University, Faculty of Pharmacy, Bucharest, Romania; 8 Centre Pasteur du Cameroun, Yaoundé, Cameroon; 9 Institut Pasteur, Unit of Hepacivirus and Innate Immunity, Paris, France; 10 Democritus University of Thrace, Department of Molecular Biology and Genetics, Alexandroupolis, Greece

## Abstract

Clinical studies have suggested association of some hepatitis C virus (HCV) subtypes or isolates with progression toward hepatocellular carcinoma (HCC). HCV core protein has been reported to interfere with host Wnt/β-catenin pathway, a cell fate-determining pathway, which plays a major role in HCC. Here, we investigated the impact of HCV core genetic variability in the dysregulation of Wnt/β-catenin pathway. We used both transient expression of core proteins from clinical isolates of HCV subtypes 1a (Cambodia), 4a (Romania) and 4f (Cameroon) and infection systems based on a set of engineered intergenotypic recombinant viruses encoding core from these various clinical strains. We found that TCF transcription factor-dependent reporter activity was upregulated by core in a strain-specific manner. We documented core sequence-specific transcriptional upregulation of several β-catenin downstream target genes associated with cell proliferation and malignant transformation, fibrogenesis or fat accumulation. The extent of β-catenin nuclear translocation varied in accordance with β-catenin downstream gene upregulation in infected cells. Pairwise comparisons of subgenotypic core recombinants and mutated core variants unveiled the critical role of core residues 64 and 71 in these dysregulations. In conclusion, this work identified natural core polymorphisms involved in HCV strain-specific activation of Wnt/β-catenin pathway in relevant infection systems.

## Introduction

Chronic hepatitis C is a slow and asymptomatic progressive disease leading to long-term complications including liver fibrosis, cirrhosis, and hepatocellular carcinoma (HCC)^1^. HCC is the second leading cause of cancer-related deaths accounting for about 800,000 deaths annually worldwide. Approximately 30% HCC cases are associated with hepatitis C virus (HCV) infection. The recent introduction of highly effective direct-acting antiviral drugs can lead to HCV clearance in over 90% of patients with advanced liver disease. However, successful HCV eradication does not eliminate the risk for HCC progression, notably in effectively treated cirrhotic patients. Consequently, in spite of efficient treatment options, HCV infection is anticipated to remain a leading cause of HCC in the next decade^2^.

HCV, as a single-stranded positive sense RNA virus replicating entirely in the cytoplasm of the host cell is unique among cancer-causing viruses. Indirect effects of chronic inflammation together with direct HCV-induced mechanisms are likely to contribute to HCV-associated HCC progression^3^. The HCV genome harbors a single open reading frame, flanked by 5′ and 3′ nontranslated regions. An internal ribosomal entry site within the 5′ nontranslated region drives the translation of the HCV genome into a single polyprotein, which is co-translationally cleaved by viral and host proteases to release ten mature proteins: core, comprising the viral particle capsid, two envelope glycoproteins, E1 and E2, and 7 nonstructural proteins, p7, NS2, NS3, NS4A, NS4B, NS5A, and NS5B^4^. A second small open reading frame within the core gene encodes an additional protein, known as ARFP or F or core+1, with as yet unknown function^5^.

Liver-specific expression of HCV full-length polyprotein or only HCV core led to liver steatosis and liver tumors in some transgenic mouse lineages^6^, pointing to a possible direct role of HCV proteins, notably of core, in hepatocellular carcinogenesis. In addition, using transient expression systems in cultured cells, HCV core has been suggested to be involved in the dysregulation of several host signaling pathways affecting transcription, apoptosis, cell proliferation, oxidative stress and lipid metabolism, all of which can lead to malignant transformation^3^. However, it is still unclear whether these regulations also occur in the course of human hepatocyte infection, likely associated with lower viral protein expression levels. Interestingly, Higgs *et al*. reported that the wingless-type MMTV integration site family (Wnt)/β-catenin signaling pathway was activated in HCV polyprotein transgenic mice, resulting in the overexpression of v-myc avian myelocytomatosis viral oncogene homolog (c-MYC) that led to increased oxidative DNA damage and impaired cell-cycle arrest^7^. In cell culture studies, the overexpression of either HCV core or NS5A of HCV subtype 1a was shown to activate the Wnt/β-catenin signaling pathway (reviewed in^8^). Many published studies emphasize the role of the Wnt/β-catenin pathway in cell fate determination, homeostasis, polarity and proliferation. In the absence of a Wnt ligand, β-catenin binds to a destruction complex and is targeted for proteasomal degradation after phosphorylation and ubiquitination. Binding of Wnt ligands to the Frizzled receptors triggers activation of the canonical Wnt/β-catenin pathway. These events prevent phosphorylation of β-catenin and result in its stabilization, cytoplasmic accumulation and subsequent translocation into the nucleus where it forms a complex with the T-cell factor/lymphoid enhancer factor (TCF/LEF) transcription factors and activates target gene expression. Mutations within this pathway, including activating mutations in the β-catenin gene, occur frequently in HCC cases, notably in HCC associated with HCV chronic infection, indicating an important role for this pathway in liver carcinogenesis (reviewed in^9^).

Virus-associated carcinogenesis has been reported to be sequence- and strain- specific for several oncogenic viruses. The most classic example is human papillomaviruses, causing contrasting risks for cervical carcinoma according to the infecting viral strains^10^. For hepatitis B virus infections, genotype C and specific viral mutations in pre-core have been associated with increased risk for HCC^11^. Several clinical studies described an association of HCV genotype 1b encoding defined residues at positions 70 (Gln) and/or 91 (Met) of core with the highest risk of HCC development^12–14^. However, model systems have been lacking to study the molecular mechanisms possibly underlying these HCV genotype/strain-specific pathogenic associations. The aim of the present study was to investigate the impact of core genetic variability on the regulation of the Wnt/β-catenin pathway first in core-expressing systems, then in infection systems. Toward that end, a panel of HCV intergenotypic recombinant viruses, that express core sequences from various clinical isolates within the backbone of a highly cell culture adapted HCV 2a strain^15^ were generated. Using these intergenotypic recombinants to infect human hepatoma cell cultures, we found that natural mutations in HCV core are involved in increased dysregulation of the Wnt/β-catenin pathway.

## Results

### HCV core proteins of various genotypes affect differentially TCF-dependent transactivation

**Figure 1:**
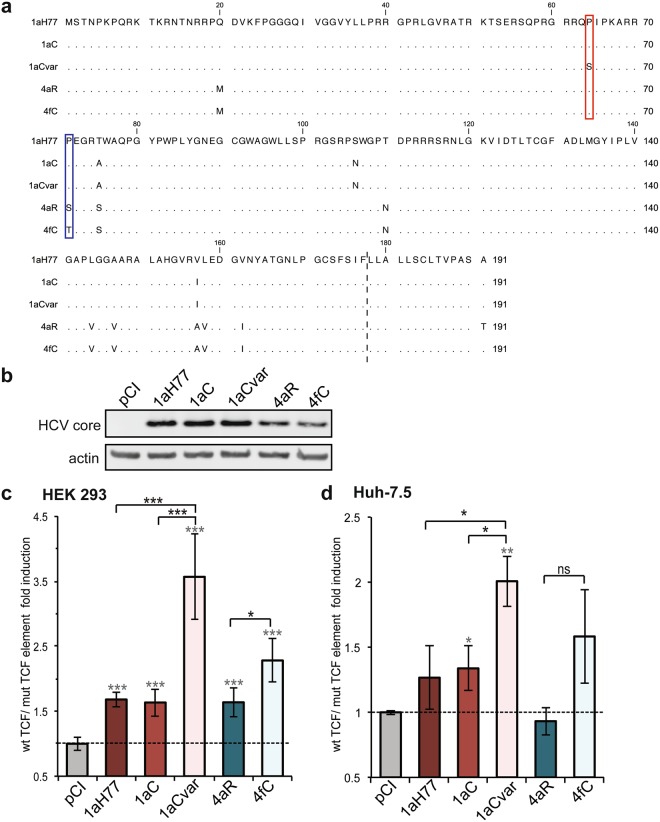
Effect of HCV core genetic variability on TCF-element dependent transcriptional activity. (**a**) An amino acid sequence alignment of core proteins from the indicated genotypes and isolates is shown with respect to HCV 1aH77 core. Dots represent identical residues. The dotted line indicates the putative C-terminus of mature core proteins according to the known 2a-JFH1 core C-terminal residue^20^ following removal of the C-terminal hydrophobic sequence by signal peptide peptidase. Red and blue boxes point to unique amino acid differences between mature core proteins of 1aC/1aCvar and 4aR/4fC variants, respectively. (**b**) HCV core expression was assessed in transiently transfected cells using anti-core and control anti-actin antibodies (pCI: empty DNA vector; full-length image of corresponding immunoblot can be found in the Supplementary information: Supplementary Fig. 5). HEK293 (**c**) or Huh-7.5 (**d**) cells were transfected with core expressing DNAs or pCI vector and either pTOP (wild-type [wt]) or pFOP (mutated [mut]) TCF element reporter DNAs. Relative wt/mut TCF element FLuc ratios are represented [means ± SD of quintuplicates obtained in 3 independent experiments (c) or of triplicates obtained in 2 independent experiments (d)]. The dotted lines indicate thresholds obtained in the absence of any core expression (*pCI*). Statistical analyses with respect to values obtained in pCI-transfected cells are indicated by grey stars above each bar (when significant), while statistical analyses between two related variants are indicated in black characters above brackets and are coded as follows: P < 0.05 (*), P < 0.005 (**), P < 0.001 (***), non-significant, P ≥ 0.05 (ns).

We examined whether HCV core proteins derived from various HCV clinical strains could potentiate host cell Wnt/β-catenin signaling activity through the modulation of TCF-dependent transcriptional activity. Core sequences from clinical strains of subtype 1a isolated in Cambodia (1aC, 1aCvar) and subtypes 4a (4aR) and 4f (4fC) isolated in Romania and Cameroon, respectively, were used in this study along with core sequence from the prototypic H77 strain of subtype 1a (1aH77). We focused on European (4aR) and Central African (4fC) HCV genotype 4 clinical strains to investigate putative differential functional properties and oncogenic potential of core proteins from emerging genotype 4 HCV strains found to circulate in Cameroon and other Central Africa countries^16^. The rationale for focusing on Cambodian 1a clinical isolates was to study the functional properties of encoded core variants in comparison with the prototype 1aH77 strain, in an effort to understand the unique core nucleotide substitutions and core antibody response found in Cambodian patients^17^. Core sequences named “1aC” and “1aCvar” were selected for further study on the basis of Tcf element differential activation (see below). Consensus, full-length core coding sequences derived from clinical isolates were determined either previously^17^ or for the purpose of this study (see Methods). A protein sequence alignment (Fig. 1a) shows that 1aC and 1aCvar core proteins differ by 3 and 4 amino acids from 1aH77 core, respectively, while 4aR and 4fC core differ by 10 and 9 amino acids from 1aH77 core, respectively.

As a first approach, HEK293 cells and human hepatoma Huh-7.5 cells^18^ were transfected with pCI-derived expression plasmid DNAs encoding core from these various isolates. All HCV core proteins were successfully expressed. Using an anti-core monoclonal antibody with cross-reactivity across various HCV genotypes, core from the three subtype 1a strains on the one hand, and from the two genotype 4 strains on the other hand, demonstrated similar expression levels (Fig. 1b). Cells were co-transfected with a reporter plasmid, pTOP^19^, containing three repeats of consensus human TCF responsive sequences upstream of the thymidine kinase promoter, which controls the transcription of firefly luciferase reporter gene (FLuc). To monitor TCF-specific effects, a FLuc reporter plasmid in which TCF responsive sequences were mutated, pFOP^19^, was co-transfected in place of pTOP. In another control, the empty vector pCI was used in place of core expressing DNA to establish basal FLuc values in this system. A third plasmid DNA, encoding β-galactosidase as a reporter protein, was also co-transfected alongside pairs of pTOP or pFOP/pCI-core DNAs in all experiments to assess transfection efficiencies and to normalize FLuc activities. Ratios of normalized FLuc activities obtained in pTOP over pFOP pCI-core-transfected cells are represented in Fig. 1c,d. In HEK293 cells, HCV core from clinical isolates 1aC and 4aR, as well as core from the prototypic 1aH77 strain demonstrated 1.5–2.0-fold higher TCF element-specific transactivation than pCI empty vector (Fig. 1c). HCV 1aCvar and 4fC core proteins showed 2.2–3.5-fold higher TCF element transactivation activities than the pCI control vector (Fig. 1c). Thus, 1aCvar core induced significantly higher TCF-dependent activation than the closely related subtype 1a C or H77 core proteins (P = 0.0009 and P = 0,0008, respectively). Likewise, 4fC core triggered significantly higher activation than the other genotype 4 (4aR) core (P = 0.0117). Similar differential transactivation activities were observed in hepatoma Huh-7.5 cells (Fig. 1d), although variations tend to be less marked, possibly due to lower transfection efficiencies than in HEK293 cells. Data from ectopic expression of this set of HCV core proteins thus suggested that amino acid polymorphisms in core may be responsible for differential TCF-dependent transcriptional modulation.

### Core amino acids Ser64 and Thr71 are responsible for the higher upregulation of TCF-element

**Figure 2:**
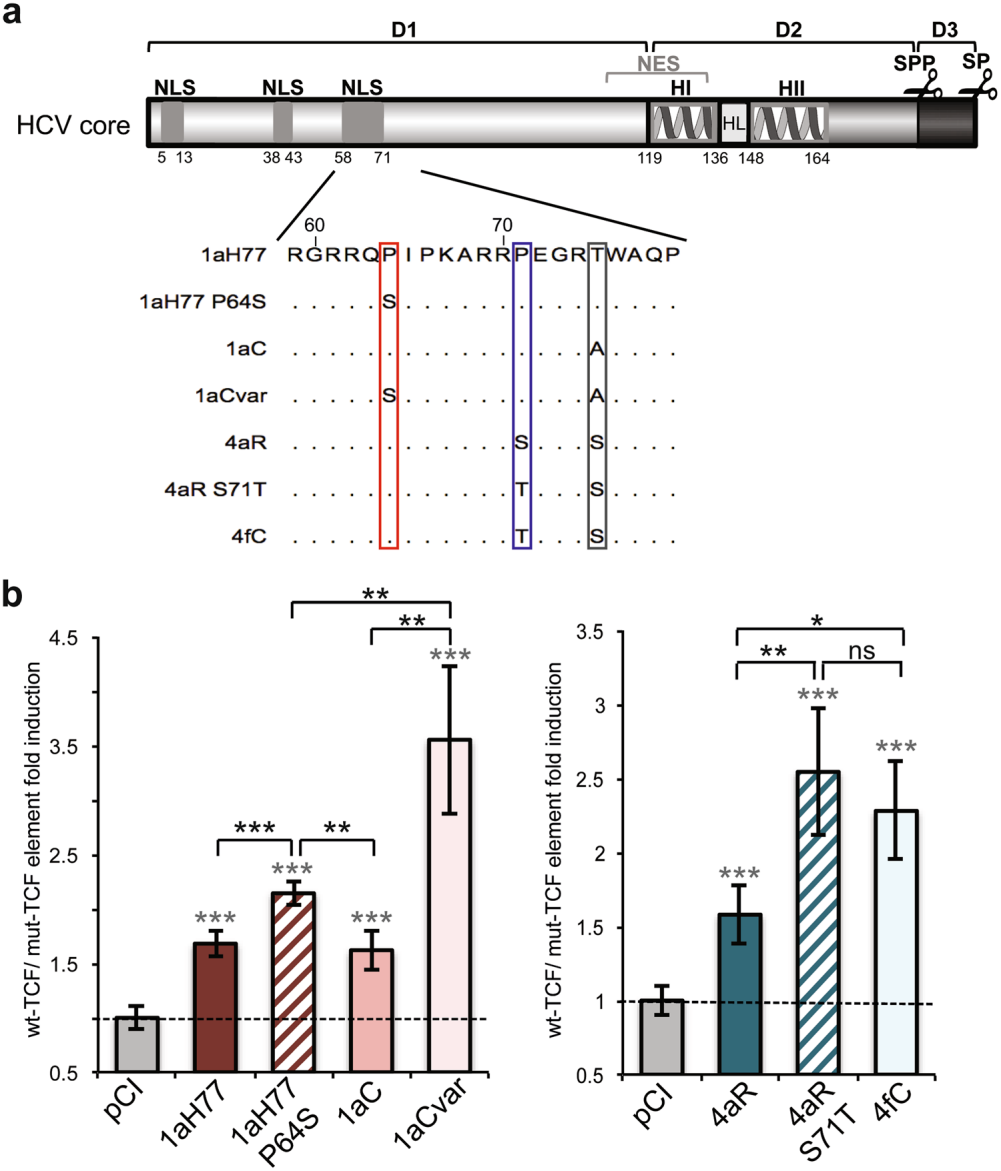
Involvement of residues at position 64 and 71 of HCV core in the modulation of TCF-element activation. (**a**) HCV core is schematically represented with its subdomains (*D1*, *D2*, *D3*) including predicted or experimentally-demonstrated nuclear localization signals (*NLS*), nuclear export signals (*NES*), alpha-helices (*H*), loop between alpha-helices (*HL*), and location of cleavage by cellular signal peptidase (*SP*) and signal peptide peptidase (*SPP*) (*scissors*). Numbering below the scheme corresponds to amino acid position framing each element in core. An alignment of core sequences from clinical isolates and engineered mutant derivatives 1aH77 P64S and 4aR S71T is shown in the blown-up below. Amino acid differences are boxed. (**b**,**c**) Relative TCF element activation in HEK293 cells expressing natural or engineered core variants and statistical analyses were established and represented as described in Fig. 1 caption (means ± SD of quintuplicates obtained in 3 independent experiments).

Mature core proteins of 1aC/1aCvar and 4aR/4fC pairs, which likely lack the 14 C-terminal amino acids following maturation by host signal peptide peptidase, according to the experimentally-determined C-terminal sequence of 2a JFH1 core^20^, differ by only one amino acid at position 64 or 71, respectively (Figs 1a and 2a). A serine residue is found at position 64 of 1aCvar core in place of Pro in 1aC and 1aH77 core, and a threonine residue is found at position 71 of 4fC core in place of Ser in 4aR core. To investigate whether these amino acid changes were associated with higher transactivation of TCF-element, we introduced site-specific mutations in 1aH77 and 4aR core coding backbones. Upon transient expression of mutated core in pTOP- or pFOP-transfected cells, we found that 1aH77 core harboring an engineered serine residue at position 64 (1aH77 P64S) induced significantly higher TCF element activation than 1aH77 or 1aC core (P = 0.0004 and P = 0.0013, respectively), both harboring Pro at this position. Upregulation by 1aH77 P64S was however not as high as that triggered by 1aCvar core which also harbors Ser64 along with 3 other substitutions (Fig. 2b). Similarly, a 4aR core derivative with an engineered S71T coding substitution (4aR S71T) led to significantly higher TCF element transactivation than 4aR core (P = 0.0035), in the same range as that observed with 4fC core (Fig. 2c). These results indicate that the nature of amino acids at positions 64 and 71 of core influences the modulation of TCF element transactivation.

### Expression of HCV core leads to strain-specific upregulation of Wnt/β-catenin downstream genes

**Figure 3:**
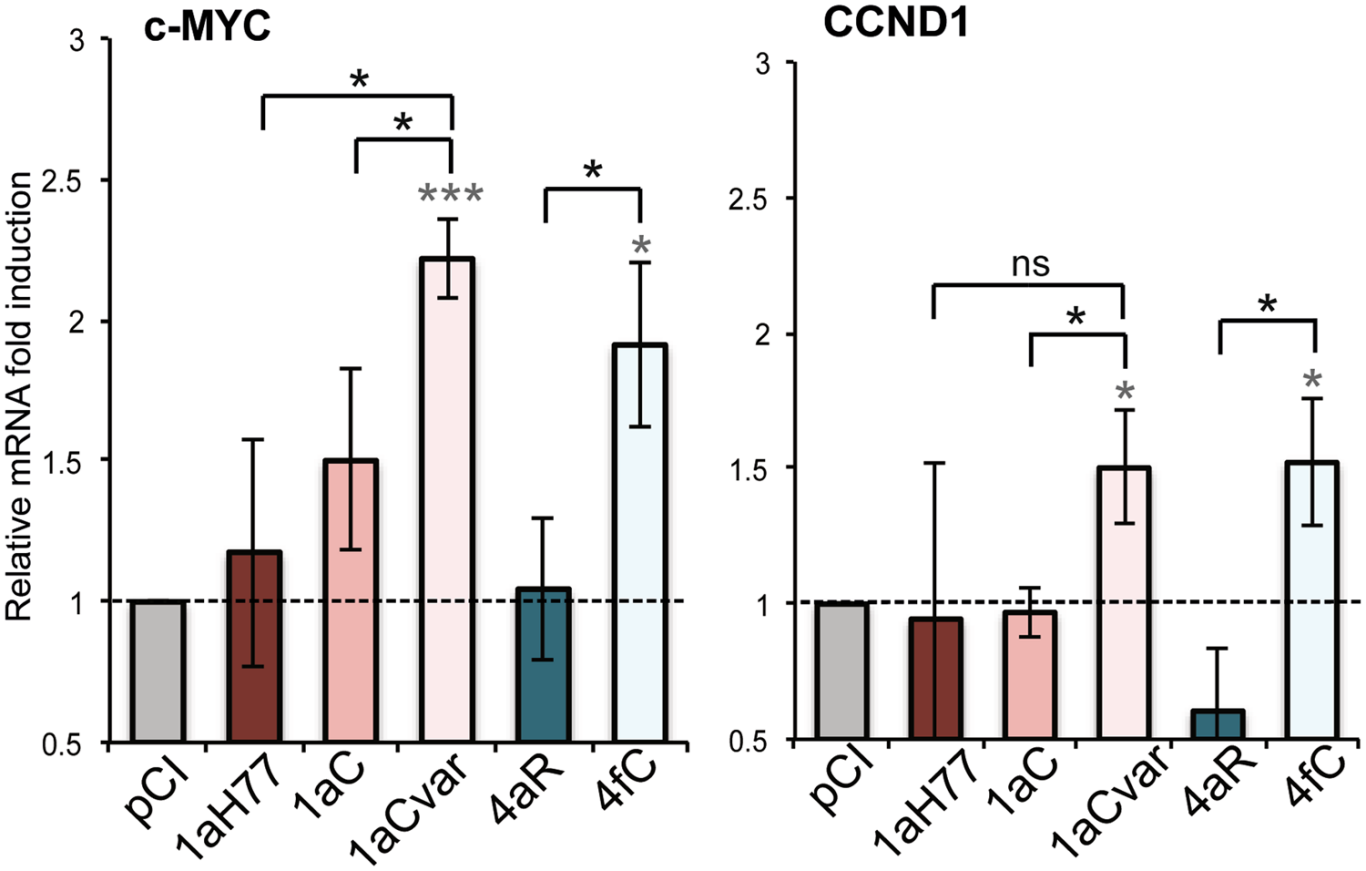
Involvement of HCV core in the induction of Wnt/β-catenin downstream genes in a variant-specific manner. mRNA levels of the genes indicated at the top of the graphs were quantified following reverse transcription and real-time quantitative PCR in HEK293 cells transfected with HCV core-expressing DNAs, then normalized with respect to housekeeping genes and expressed relatively to respective target mRNAs in pCI-transfected cells, set at 1 (means ± SD of 3 independent experiments). Statistical analyses are as described in Fig. 1 caption.

To further investigate the role of core variants in the regulation of the Wnt/β-catenin signaling pathway, we assayed the effect of core expression on the transcriptional regulation of genes associated with cell proliferation and survival, which are known downstream targets of the Wnt/β-catenin signaling pathway^21,22^. For this purpose, we quantified c-MYC and Cyclin D1 (CCND1) mRNA levels in transfected cells. These target gene mRNA levels were normalized with respect to GAPDH as a housekeeping gene (see Methods). As shown in Fig. 3, 1aCvar and 4fC core variants induced a 1.5–2.2-fold transcriptional upregulation of c-MYC and CCND1. In contrast, 1aH77, 1aC and 4aR core variants did not significantly affect these genes. Significant differences were observed in gene upregulation levels between C(4aR)- and C(4fC)-expressing cells (P = 0.0333 and P = 0.0172 for c-MYC and CCND1, respectively), as well as between C(1aC)- and C(1aCvar)-expressing cells (P = 0.0449 and P = 0.0302). These results indicate that, in agreement with TCF transactivation data, HCV core upregulates known Wnt/β-catenin downstream genes in a core sequence-specific manner.

### Regulation of Wnt/β-catenin downstream genes by HCV core variants in infection systems

**Figure 4:**
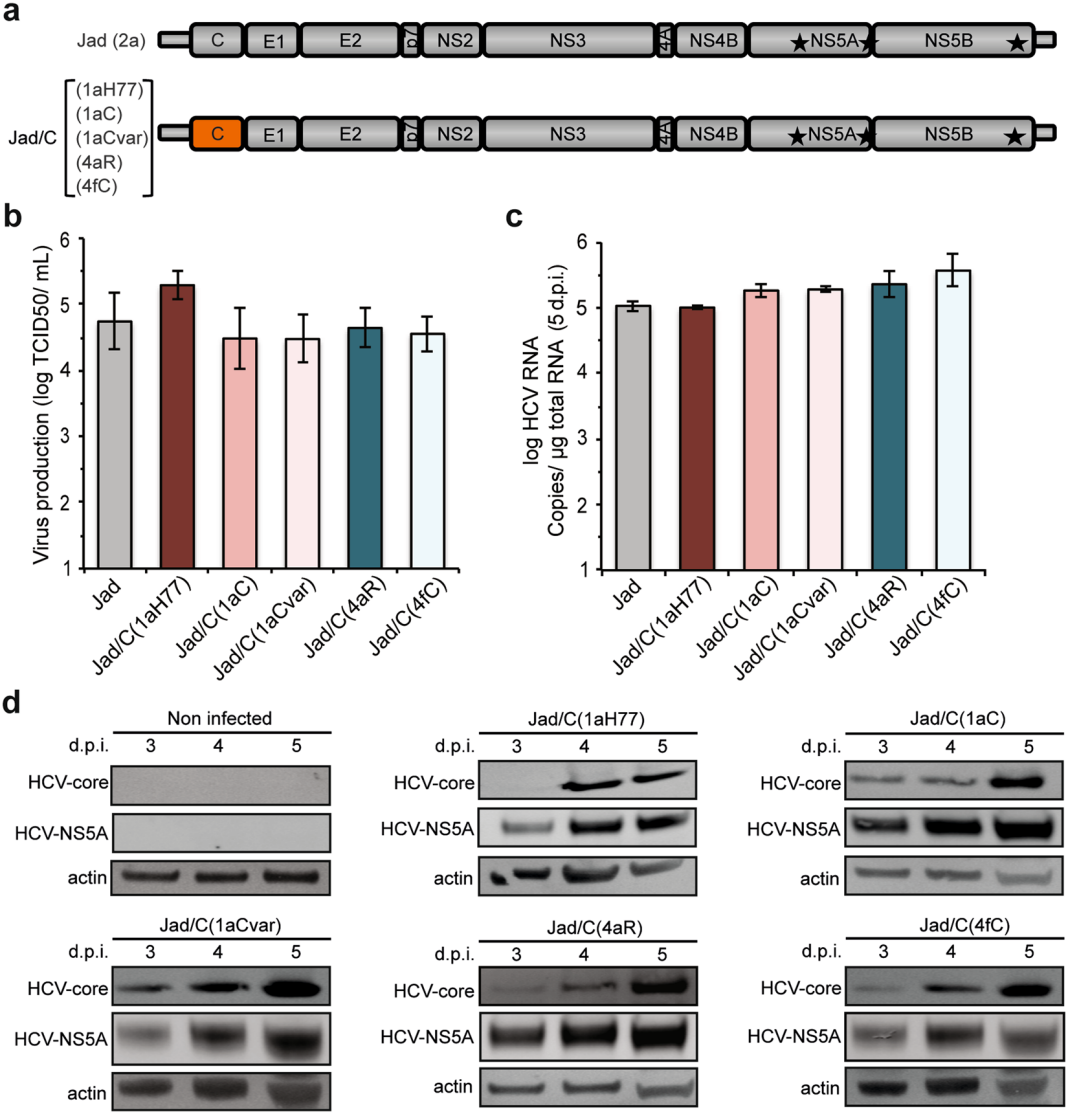
Generation and characterization of intergenotypic core recombinant viruses. (**a**) Schematic representation of intergenotypic Jad(2a) recombinant cDNAs encoding core of the indicated genotypes and strains. (**b**) Infectious titers obtained at 3 days post-transfection with intergenotypic core recombinant RNAs (means ± SD of 4 transfections). (**c**) Intergenotypic chimeric RNA abundance was quantified in infected Huh-7.5 cells at 5 days post-infection by reverse transcription-quantitative PCR (means ± SD of 2 experiments). (**d**) Viral protein expression was monitored in infected Huh-7.5 cells at 3, 4, and 5 days post-infection using anti-HCV core and anti-HCV NS5A antibodies, as well as control anti-actin antibodies. Full-length images of corresponding immunoblots can be found in the Supplementary information (Supplementary Fig. 6).

Transient expression systems as described above could lead to protein overexpression that may trigger non-biologically relevant cellular dysregulations. Therefore, it was essential to assess the sequence-specific effect of HCV core variants on the Wnt/β-catenin signaling pathway in the context of a relevant HCV infection model. Most existing HCV infection systems rely on Huh-7.5 cell line and Japanese fulminant hepatitis 1 strain (JFH1) of HCV genotype 2a^23^ or cell culture-adapted intergenotypic recombinants encoding proteins associated with viral morphogenesis (core C, envelope glycoproteins E1/E2, ion channel protein p7, and nonstructural protein NS2) from a non-2a genotype connected to JFH1 nonstructural proteins NS3 to NS5B^24,25^. Here, we engineered core intergenotypic recombinant cDNAs within the backbone of a robust cell culture-adapted derivative of HCV JFH1 (Jad)^15,26^ by replacing only the core coding sequence of JFH1 by that of 1aH77, 1aC, 1aCvar, 4aR, and 4fC (Fig. 4a).

The transfection of Huh-7.5 cells with corresponding synthetic Jad core recombinant RNAs gave rise to robust progeny viruses for all core sequences with similar titers (~5 × 10^4^ TCID50/mL at 3 days post-transfection) (Fig. 4b). Recombinant virus stocks were produced with infectious titers in the 5 × 10^5^–1 × 10^6^ TCID50/mL range.

To evaluate the effect of core genetic variability on the Wnt/β-catenin pathway in the context of HCV infection, Huh-7.5 cells were infected at high multiplicity of infection (10 TCID50/cell) with intergenotypic recombinant viruses encoding core from the 1aH77, 1aC, 1aCvar, 4aR, and 4fC strains described above. The transcriptional regulation of c-MYC and CCND1, as well as other cellular genes known to be upregulated upon activation of the Wnt/β-catenin pathway and to be associated with chronic HCV disease manifestations [T-box 3 (TBX3), axin 2/conductin (AXIN2), fibronectin type III domain containing 3B (FNDC3B), fatty acid synthase (FASN)] was monitored in infected cells at 5 days post-infection. This time point was chosen since all recombinant core viruses demonstrated high and similar viral RNA abundance (Fig. 4c). HCV protein levels (exemplified by NS5A and core detection) also peaked at 5 days post-infection and were very similar across groups of subtype 1a and genotype 4 viruses that were compared (~12% and 16% protein level variations, respectively, Fig. 4d). In addition, core proteins from all genotypes examined co-localized with lipid droplets, as a hallmark of HCV infection (Supplementary Fig. 1). Together, these features demonstrated the functionality of all core variants expressed from recombinant viruses. Of note, “minicore” polypeptides corresponding to C-terminal core sequences have been reported to be expressed by internal initiation (notably p8 from amino acid 91 but also larger forms), along with full-length core of genotype 1 or 2a HCV^27^. Using polyclonal antibodies raised against amino acid 1–120 of HCV 1aH77 core protein, thus expected to react with both N- and C-terminal epitopes of core^17^, we did not detect shorter immuno-reactive products that could correspond to “minicore” proteins in cells infected with Jad or the various core recombinants (Supplementary Fig. 2).

**Figure 5:**
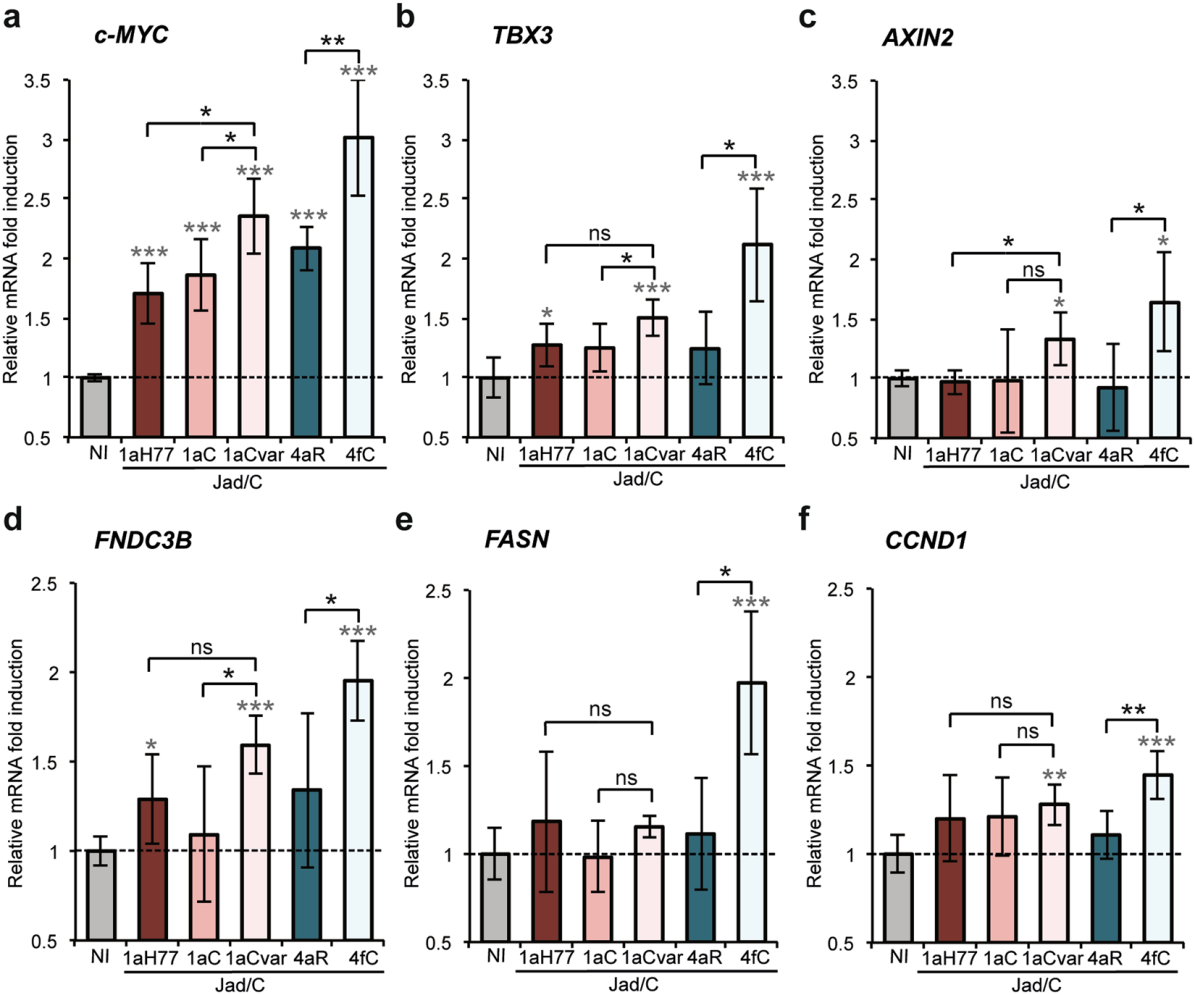
Transcriptional regulation of Wnt/*β*-catenin pathway downstream genes in infected cells. Following infection with the indicated viruses, levels of c-MYC (**a**), TBX3 (**b**), AXIN2 (**c**), FNDC3B (**d**), FASN (**e**) and CCND1 (**f**) mRNAs quantified at 5 days post-infection were normalized with respect to housekeeping genes and expressed with respect to the relative abundance of respective mRNAs in non infected (NI) cells, set at 1. Means ± SD of sextuplicates obtained in 4 independent experiments. Statistical analyses are as described in Fig. 1 caption.

Levels of Wnt/β-catenin target gene mRNAs were determined in infected cells following reverse transcription and real-time quantitative PCR (qPCR) with respect to a set of 4 housekeeping genes (see Methods) (Fig. 5). We found that infection with Jad/C(4fC) resulted in the significant upregulation of c-MYC (P < 4 × 10^−6^, Fig. 5a), TBX3 (P < 0.0006, Fig. 5b), AXIN2 (P < 0.0068, Fig. 5c), FNDC3B (P < 1 × 10^−5^, Fig. 5d), FASN (P < 0.0005, Fig. 5e) and CCND1 (P < 0.0002, Fig. 5f), with c-MYC and TBX3 showing the highest upregulation levels. In addition, the gene upregulation induced by Jad/C(4fC) was significantly higher than that induced by Jad/C(4aR) (Fig. 5a–f with P values < 0.0028, 0.0061, 0.017, 0.037, 0.0071, 0.0031, respectively), confirming that the nature of amino acid 71 is an important player in this regulation. Similarly, although less pronounced, Jad/C(1aCvar) induced significant upregulation of all genes (P < 3 × 10^−6^, 0.0006, 0.0084, 7 × 10^−5^, 0.0024, respectively in Fig. 5a–d and f) except FASN (P = 0.0749) (Fig. 5e). Activation by Jad/C(1aCvar) was substantially higher than that induced by Jad/C(1aC) infection, highlighting also the role of residue 64 in infection models.

### Nuclear accumulation of β-catenin in cells infected with core recombinant viruses varies according to core sequences

**Figure 6:**
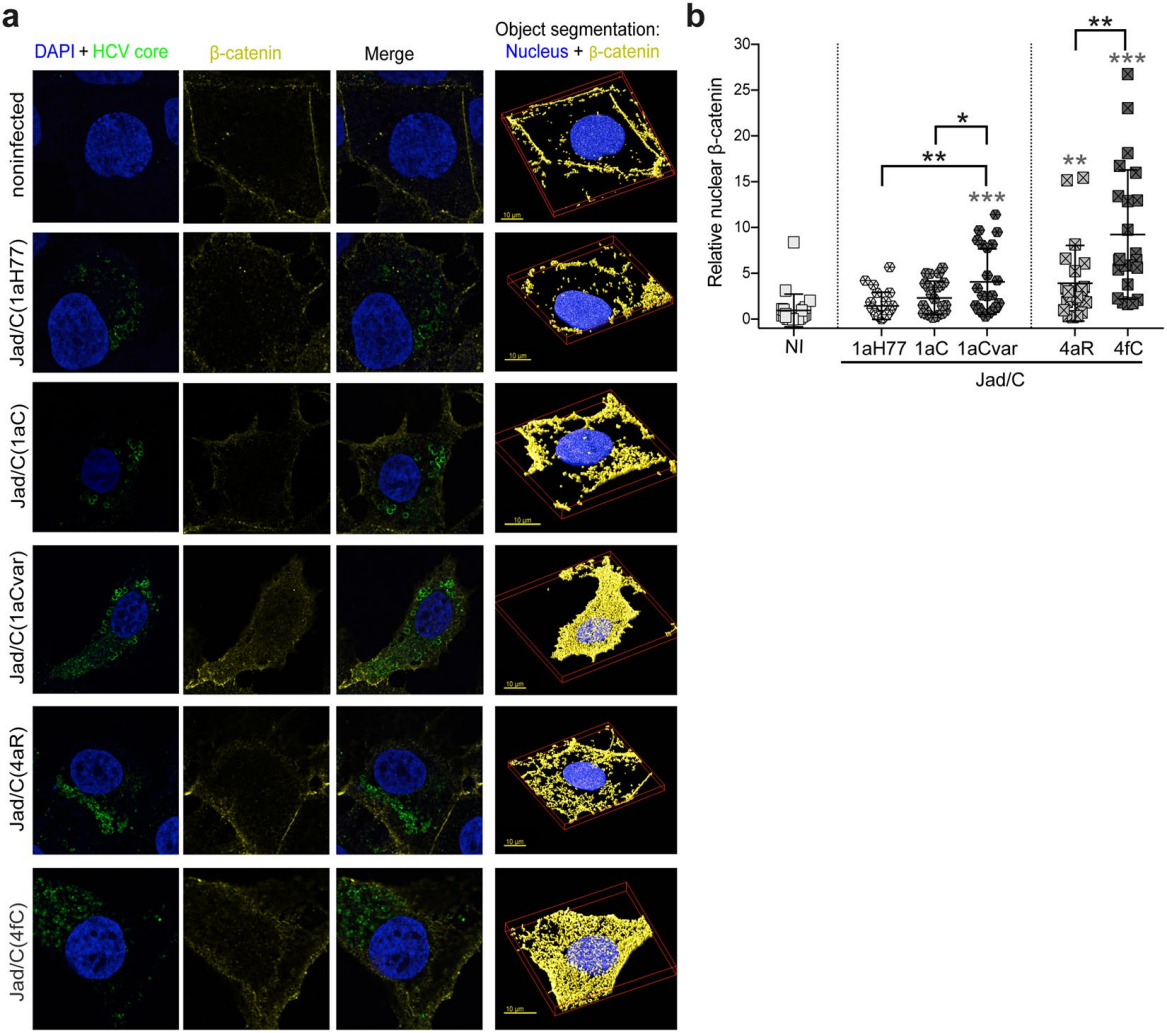
Strain-specific, core-dependent nuclear translocation of *β*-catenin in infected cells. (**a**) Huh-7.5 cells infected with the indicated viruses or noninfected cells were labeled for nucleus (DAPI, blue) and core (green), as well as for β-catenin (yellow). Merged deconvolved images and representative 3D segments used for object segmentation (nuclei and β-catenin, right images) are shown. (**b**) Images were subjected to object analysis (co-localization intersection) using Huygens Professional software and intersecting volumes between β-catenin and nucleus were quantified per cell. Intersecting voxels (means and distribution among 23 cells per condition, each cell being represented by a symbol) were expressed relatively to intersecting voxels found in noninfected cells set at 1. Statistical analyses with respect to values obtained in noninfected cells are indicated above each group of virus-infected cells (in grey characters), while statistical analyses between two related variants are indicated in black characters above brackets. These statistical analyses were performed according to the Holm-Sidak method and are coded as follows: P < 0.05 (*), P < 0.005 (**), P < 0.001 (***).

A hallmark of Wnt/β-catenin signaling pathway consists in β-catenin nuclear translocation, which results in turn in the activation of target genes^9^. We thus examined by confocal microscopy the cellular distribution of β-catenin in cells infected with intergenotypic chimeras Jad/C(1aH77), Jad/C(1aC), Jad/C(1aCvar), Jad/C(4aR) and Jad/C(4fC). Infected cells were labeled for HCV core and β-catenin with appropriate antibodies, and cell nuclei were labeled with 4-6-diamidino-2-phenylindole (DAPI). Z-stacked images of infected cells were acquired for each virus along with images of noninfected cells (23 images per condition). Representative deconvolved images of single cells depicting labeling of nucleus/HCV core (left), β-catenin (middle), and merged images (right), as well as 3D-rendered DAPI/β-catenin labeling are shown in Fig. 6a. Magnification of 10 randomly-picked, 3D-rendered images centered on cell nuclei are shown in Supplementary Fig. 3. Deconvolved 3D images centered on single cells were analyzed using SVI-Huygens software following object segmentation that allows the scoring of total volumes of distinct labeled channels within a cell, as well as intersecting volumes between two channels, as detailed in the Methods. Quantification of total β-catenin present at the cell membrane, as well as within the cytosol and the nucleus of each infected or noninfected cell showed slightly [Jad/C(1aH77)] or significantly [Jad/C(1aC), Jad/C(1aCvar), Jad/C(4aR), p < 0.05; Jad/C(4fC), p < 0.001] higher accumulation of total β-catenin in infected cells than in noninfected cells. In addition, there was significantly higher (p < 0.05) total β-catenin abundance in Jad/C(4fC) than in Jad/C(4aR) infected cells. Differences in total β-catenin abundance were more modest between Jad/C(1aCvar) and Jad/C(1aC) infected cells (Supplementary Fig. 4). Importantly, quantification of intersecting volumes of β-catenin within the nucleus revealed significantly higher nuclear accumulation of β-catenin in cells infected with Jad/C(4fC) than in cells infected with Jad/C(4aR) (P < 0.005, Fig. 6b). Similarly, significantly higher nuclear abundance of β-catenin was observed in cells infected with Jad/C(1aCvar) than in cells infected with either Jad/C(1aC) or Jad/C(1aH77) (P < 0.05 and P < 0.005, respectively, Fig. 6b). These important findings corroborate the HCV strain-specific transcriptional upregulation of the Wnt/β-catenin downstream genes and point to the role of natural core polymorphisms in triggering this pathway. These results also support the role of β-catenin in the activation of Wnt/β-catenin downstream genes.

## Discussion

Mechanisms involved in HCV-induced liver carcinogenesis are still unclear. The Wnt/β-catenin signaling pathway involved in hepatic cell homeostasis, liver regeneration but also in HCC development has been shown to be activated by HCV^8^.

In this study, we initially showed that the transient expression of HCV core from the prototypic strain H77 of subtype 1a, but also from other 1a clinical strains (1aC, 1aCvar) mediated the upregulation of TCF element activation in HEK293T cells (Fig. 1c). These data extended findings from Liu *et al*. who reported TCF element upregulation in HCV 1aH77 core overexpression systems^28^. Importantly, we found a strain-dependent regulation of TCF element among genotype 1 or 4 clinical strains, both in HEK293T and Huh-7.5 cells that transiently express core (Fig. 1c,d). Although core is relatively conserved across HCV genotypes, natural mutations specifically occurring within the core coding region have been reported in virus isolated from liver tumor tissue^29^. From clinical studies, some polymorphisms in core have been associated with enhanced potential of HCV 1b chronic infections to progress towards HCC, notably amino acid mutations at position 70 of core^13,14,30^. Here, by comparing the impact of core from various strains, we unveiled the importance of other amino acid residues located in the same core region, namely at position 64 in the context of HCV 1a core and at position 71 in the context of HCV 4 core, which were responsible for the varying transactivation levels of TCF element (Fig. 2b,c). Consistent with these observations, we found that several genes downstream of the Wnt/β-catenin pathway that are critical for the regulation of cell cycle and tumorigenesis were transcriptionally activated by the core variants demonstrating the highest TCF element upregulating properties. Importantly, variations of these gene transcriptional levels were similarly observed both in core transient expression systems and in the more relevant system based on the infection with cognate core recombinant viruses (Figs 3 and 5). It should be stressed that fold-inductions of Wnt/β-catenin target genes, although relatively modest, were reproducible across experiments, statistically-significant, and in the range of those described in HCV core transient expression systems^28^ or upon other viral infections in cell culture and in murine models (e.g. with Rift Valley Fever virus), for which they were shown to be biologically-relevant^31^. Accordingly, recombinant viruses that express core variants with the highest gene upregulation activities [(Jad/C(1aCvar), Jad/C(4fC)] induced increased nuclear relocalization of β-catenin (Fig. 6), corroborating the activation of the Wnt/β-catenin canonical pathway. HCV 1aH77 NS5A was also reported to activate Wnt/β-catenin pathway via a novel phosphoinositide-3 kinase-mediated mechanism that involves direct interaction between NS5A and β-catenin^32^. Chimeric viruses generated in our study encode identical NS5A, enabling the specific monitoring of the effects induced by divergent core species on Wnt/β-catenin pathway target genes. Shorter “minicore” proteins resulting from internal initiations within the core coding sequence have been described for 1b-Con1 and 2a-JFH1 strains^27^. In addition, core+1/ARFP proteins resulting from different translation mechanisms operating in an overlapping ORF within HCV core coding sequence have also been documented, essentially for genotypes 1 and 2. Internal initiation at core codons 25–26 or 85–87 have been reported to be involved in expression of long and short forms of core+1/ARFP, respectively (reviewed in^33^). Thus, functional properties attributed to core might theoretically be alternatively or in addition attributed to minicore or core+1 proteins produced along with core. We did not detect shorter forms of core in cells infected with the core recombinant viruses using polyclonal anti-core antibodies directed against ~70% of core (aa 1–120) expected to detect such forms (Supplementary Fig. 2). Although evidence has been provided for the presence of core+1-specific antibodies in HCV infected patients, core+1 detection in transfected or infected cells using specific antibodies has not been possible unless reporter tag fusion strategies were used, likely due to protein intrinsic instability^5^. Therefore, we could not reliably address the question of putative core+1 expression in cells infected by core recombinants. It should be underscored, however, that, if core+1 was expressed from the variants described in our study, the nucleotide change at position 64 of subtype 1a strains would not introduce an amino acid change in the core+1 ORF. Should core+1 production occurs in genotype 4 strains and initiates at similar sites as for genotypes 1 and 2, we shall stress that the nucleotide substitution at core codon 71 (subtypes 4a/4f) results in a conservative aspartate toward glutamate change in the amino acid sequences of core+1 ORF. This would be expected to impact only minimally the putative long form of core+1. Altogether, these observations indicate that the phenotypes observed for core variants described in this study are likely linked to full-length core proteins only.

Among the known downstream targets of Wnt/β-catenin that were upregulated to higher levels in Jad/C(1aCvar) and Jad/C(4fC) infected cells, c-MYC and Cyclin D1 (CCND1) are associated with malignant cell transformation and progression to HCC^34^. Our data are consistent with the upregulation of β catenin-dependent c-MYC transcription in an HCV polyprotein transgenic mouse model^7^ and suggest that core triggers this upregulation. Our data are also consistent with increased Cyclin D1 and c-MYC protein levels that were documented in hepatic cells through Wnt/β-catenin signaling activation in response to HCV-induced expression of miRNA155, a marker of HCV infection in patients^35^. AXIN2 is a scaffold protein involved directly in the Wnt/β-catenin pathway that has been reported to be mutated in some cases of HCC^36^. In addition, AXIN2 is induced in response to increased β-catenin levels and promotes phosphorylation of β-catenin by Glycogen synthase kinase 3 (GSK3), thereby targeting it to degradation. AXIN2 thus serves as a negative regulator of the Wnt/β-catenin signaling pathway^37^. Our results showing transcriptional upregulation of AXIN2 (Fig. 5c) suggest the activation of this negative feedback loop during infection with Jad/C(1aCvar) and Jad/C(4fC). In spite of this negative feedback activation, the signaling pathway is not fully restrained, as indicated by core-mediated upregulation of c-MYC, TBX3, Fibronectin, FASN and Cyclin D1 (Fig. 5a–b and d–f), as well as β-catenin abundance in the nucleus (Fig. 6). It is mechanistically plausible that AXIN2 acts only on the cytosolic fraction of β-catenin, leading to its phosphorylation and degradation, while β-catenin that had translocated into the nucleus would escape this negative feedback loop. TBX3 is a downstream target gene and a critical mediator of the Wnt/β-catenin signaling pathway and is involved in proliferation and survival of liver cells^38^. TBX3 overexpression is well-documented in liver tumorigenesis^38^. Therefore, the HCV core-dependent differential regulation of c-MYC, Cyclin D1, AXIN2, and TBX3 provides one likely mechanism involved in HCV strain-dependent HCC development.

Fibronectin (FNDC3B) is an extracellular matrix protein, which accumulates during fibrogenesis. Interestingly, fibronectin level has been found to exhibit a clinical diagnostic value for liver fibrosis prediction in chronic hepatitis C infection^39^. The HCV core-dependent FNDC3B regulation that we observed may be directly responsible for HCV-induced fibrosis. We also showed that fatty acid synthase (FASN) transcription is significantly upregulated in Jad/C(4fC) infected cells (Fig. 5e). FASN transcription upregulation has been observed in cells infected with HCV-2a JFH1 and postulated to be involved in HCV-induced steatosis^40^. Importantly, our observations are in line with the fact that fatty acid biosynthesis is hyperactivated in various tumors and that FASN is the major metabolic enzyme responsible for fatty acid synthesis, which also synergizes with Wnt-mediated signal transduction to β-catenin^41,42^.

Natural mutations in the core coding sequence of clinical isolates 1aCvar and 4fC that are responsible for the significant activation of TCF element (Fig. 2) and in turn of c-MYC, TBX3, AXIN2, FNDC3B and CCND1 genes (Figs 3 and 5) have been described in other strains of HCV genotypes 1 and 4. Residue Thr71 has been reported in 18 isolates of HCV genotype 4 in Gabon^16^ and Ser64 natural mutation has been reported in a limited number of HCV genotype 1 clinical isolates^43,44^. Residues 64 and 71 are located in the hydrophilic and basic domain of mature core protein (domain D1, amino acids 1–118, Fig. 2a). Core domain D1 is predicted to be unfolded and is involved in RNA binding and homotypic interactions^45^, as well as interactions with NS5A that are critical for viral particle assembly^46^. Interestingly, although HCV life cycle is cytoplasmic, HCV-1a core was shown to contain several nuclear localization signal sequences (NLS) and nuclear export signals that are functional to bind cargo proteins and cross the nuclear pore complex^47^. Core residues at positions 64 and 71, which are the targets of the critical natural mutations identified in this study are part of the third NLS (NLS3 58-71aa, Fig. 2a). HCV core was reported to physically interact with nucleoporin 98^20^. It has been postulated that HCV hijacks nuclear transport factors and nucleoporins within the virus-induced membranous web to help compartimentalize HCV replication and assembly^20,47^, thereby possibly resulting in host signaling pathway dysregulation. Interestingly, core Gln70 has been suggested to be an important determinant of HCC development in patients infected with subtype 1b strains^12–14^. It would be valuable to generate intergenotypic recombinant viruses expressing core from various 1b strains and investigate whether Wnt/β-catenin or other host signaling cascades involved in liver carcinogenesis may be differentially impacted in cells infected with such recombinants.

Altogether, our study relying on intergenotypic core recombinant viruses provides insight into the molecular mechanisms of HCV strain-specific association with HCC, highlighting the importance of the Wnt/β-catenin cellular pathway and the role of natural mutations P64S and S71T of HCV core in the upregulation of this pathway. It is tempting to speculate that a core region comprising residues 64 and 71 may be involved in the interaction with cellular factors modulating the Wnt/β-catenin pathway activation. Further studies are required to identify the potential isolate-specific core interactors involved in these regulations. This study also highlighted the usefulness of a panel of intergenotypic core recombinant viruses that can be utilized to investigate the effect of genotype-specific core determinants on cellular signaling pathways driving HCV-induced clinical outcomes in relevant established cell lines, primary cells, or murine animal models.

## Methods

### HCV field strains

A field strain of HCV subtype 1a (designated 1aC) and a variant of this strain (designated 1aCvar), collected in Cambodia in a blood donor, were generously provided by A. Sall (Institut Pasteur du Cambodge, Phnom Penh, Cambodia). A subtype 4a strain (designated 4aR) was collected in Romania in a blood donor and a subtype 4f clinical strain (designated 4fC) was collected in a HCC patient in Cameroon. Consensus HCV core coding sequences from the field strains were obtained following RNA extraction from patient plasma samples, reverse transcription then PCR amplification using primer pairs designed to hydridize genotype-matched 5′ nontranslated region and E1 coding sequences (Supplementary Table 1), and cloning as previously described^17^. Five clones were sequenced for each strain using capillary eletrophoresis (CeMIA, Eurofins), from which consensus core coding sequences were established. HCV core sequences reported in the present study have been submitted to European Nucleotide Archive (ENA) and have been assigned accession numbers ERZ664228, ERZ655053, ERZ655054 and ERZ672786.

### Ethics statement

This study conformed to the ethical guidelines of the 1975 declaration of Helsinki and was approved by either the Cameroon National Ethics Committee (Number 199/CNE/SE/2011) and the Ministry of Health of Cameroon (Number 631–01.12), the National Ethics Committee for Health Research at Ministry of Health of the Kingdom of Cambodia (Number 1296/02 OGH) and by the Pasteur Institute of Cambodia, or the Bioethics Committee of the Cantacuzino National Medical-Military Institute of Research and Development from Romania (Number 16/CE/26.11.2012). Written informed consent was obtained from all patients.

### Cells

Human embryonic kidney 293 (HEK293) cells were cultured in Dulbecco’s modified Eagle’s medium (Life Technologies), supplemented with 10% fetal calf serum, 100U/ml penicillin and 100 mg/ml streptomycin (DMEM-10%) at 37 °C in a 5% CO_2_ atmosphere. Human hepatoma Huh-7.5 cells^18^ (kindly provided by C. Rice) were cultured in DMEM-10% supplemented with nonessential amino acids and 1 mM sodium pyruvate (complete DMEM).

### Plasmids

Genome-length cDNA of the prototypic H77 strain of HCV subtype 1a (1aH77, GenBank accession #AF009606)^48^ was generously provided by C.M. Rice (The Rockefeller University, New York, USA). Core coding sequences were cloned into the mammalian expression vector pCI (Promega), as previously described^17^, generating pCI/core -1aH77, -1aC, -1aCvar, -4aR, 4fC plasmids. Wild-type or mutated Tcf4-responsive luciferase reporter pTOP and pFOP plasmids^19^ and pA-EUA2 + lacZ plasmid were kindly provided by C. Neuveut (Institut Pasteur, Paris, France) and A.L. Epstein (University Claude Bernard, Lyon, France), respectively. Nucleotide substitutions encoding amino acid mutations P64S (amino acid numbering according to position within core; C530T, nucleotide numbering according to genome-length cDNA position) or S71T (T551A) were introduced into pCI/core-1aH77 and pCI/core-4aR, respectively, using QuickChange Site-Directed Mutagenesis Kit (Agilent Technologies, Santa Clara, USA) and appropriately designed primers. Plasmid pJad^26^ was derived from pJFH1 (genome-length HCV cDNA of subtype 2a)^23^ and contains cDNA substitutions resulting in three amino acid changes that confer high-titer cell culture adaptation^15^. Plasmids pJad/C(1aH77), /C(1aC), /C(1aCvar), /C(4aR), and /C(4fC) were generated by an overlapping-PCR strategy^24^ designed to substitute Jad 2a core coding sequence (nucleotides 341–913 of the genome length cDNA) by core sequences of the indicated HCV strains. The resulting PCR fragments contained unique *Age*I and *Bsi*WI restriction sites at their 5′ and 3′ extremities, respectively, which were used for cloning into pJad at the corresponding sites. The sequences of all PCR-amplified DNA fragments from selected plasmid clones were confirmed by nucleotide sequencing. Details of all constructions can be obtained upon request.

### DNA transfection and firefly luciferase assays

HEK293 or Huh-7.5 cells were seeded at 8 × 10^4^ or 5 × 10^4^ cells per well, respectively, into 48-well plates and co-transfected with 0.2 µg pTOP (wild-type [wt] TCF element) or pFOP (mutated [mut] TCF element) reporter plasmid DNA expressing firefly luciferase (FLuc), 0.05 µg pA-EUA2 + lacZ plasmid DNA, and 0.3 µg pCI/core or pCI plasmid DNA by using jet PEI (Polyplus Transfection, Illkirch, France). Quantification of FLuc (relative light units) and β-galactosidase levels in transfected cell extracts prepared at 2 days post-transfection were performed by using Luciferase Assay System and Beta-Glo Assay System kits (Promega, Madison, Wisconsin, USA) according to the supplier’s instructions. For each sample, relative light units were normalized with respect to the β-galactosidase activity as an indicator of the transfection efficiency and expressed as ratios of activities found in pTOP over pFOP transfected cells with respect to ratios observed in pCI transfected cells set at 1 (wt TCF/mut TCF element fold induction).

### *In vitro* transcription and RNA transfection

Genome-length Jad and intergenotypic Jad/C recombinant cDNAs were linearized with *Xba*I and treated with mung bean nuclease (New England BioLabs, Evry, France) prior to *in vitro* transcription using T7 RiboMAX Express Large Scale RNA production system (Promega) and purification of resulting synthetic RNAs, as described previously^49^. Huh-7.5 cells (2 × 10^6^ cells) were transfected by electroporation with 5 µg of synthetic, genome-length RNAs, in 4-mm-gap-width cuvettes by applying one pulse at 240 V at 900 F using EasyjecT Plus instrument (Equibio, Lancashire, United Kingdom). Electroporated cells were then immediately resuspended in complete medium and seeded at 1.6 × 10^6^ cells per 75 cm^2^ flask.

### Preparation of HCV stocks and HCV TCID50 titration

Large volumes of HCV stocks were prepared following infection at a multiplicity of infection (MOI) of 0.01 50% tissue culture infectious doses 50 (TCID50) per cell with supernatants collected post-RNA transfection. Infected cells were generally split at 3 and 5 days post-infection and supernatants were collected at 5 and 7 days post-infection, clarified by a 1000 g centrifugation for 5 min at 4 °C and stored at −80 °C in small aliquots. To determine virus stock infectious titers, TCID50 assays were performed. For this, Huh-7.5 cells seeded at a concentration of 4.5 × 10^3^ or 2.5 × 10^3^ cells per well in 96-well plates were infected 24 h later with 5-fold serial dilutions of transfected or infected cell supernatants. At 3 or 5 days post-infection, respectively, cells were fixed with methanol, then processed for detection of infected cell foci and calculation of viral titers (TCID50/ml) as described previously^26,49^.

### Quantification and sequence analysis of mRNAs and viral RNAs

Total RNA was isolated from virus infected cells at 5 days post-infection and from core-expressing DNA transfected cells at 48 h post-transfection using RNAII kit (Macherey-Nagel, Düren, Germany), RNeasy Micro Kit (Qiagen, Courtaboeuf, France) or by extraction with TRIzol (Life Technologies), in accordance with the manufacturers’ instructions, and quantified by optical density measurements. Total RNA (1 μg) was reverse transcribed using Moloney Murine Leukemia Virus Reverse Transcriptase (M-MLV, Promega) or SuperScript II Reverse Transcriptase (ThermoFisher, Waltham, USA) and a random-sequence hexamer primer (d(pN)6, Roche Diagnostic, Meylan, France) or an oligo(dT) primer (ThermoFisher) according to manufacturers’ instructions. Resulting cDNAs were amplified by real-time quantitative PCR using GoTaqPCR Master Mix (Promega) or FastStart SYBR Green Master (Roche Diagnostic) and primers listed in Supplementary Table 2. For normalization purposes, geometric means of mRNA levels of SFRS4, GAPDH, HMBS164, and BC002942 housekeeping genes were quantified in Huh-7.5 cell extracts^50,51^, while GAPDH gene was used in HEK293 cell extracts. Viral RNA was quantified by one step reverse transcription-quantitative PCR using 20 ng of total intracellular RNA and TaqMan RNA-to-CT™ 1-Step Kit (Applied Biosystems) with primers and probe targeting the HCV 5′ nontranslated region as described previously^24^. Viral RNA levels were normalized with respect to 18S RNA levels quantified in parallel using TaqMan ribosomal RNA control reagents (Applied Biosystems). For sequencing purposes, viral RNA extracted from RNA-transfected or virus-infected cells was converted to cDNA using SuperScript II reverse transcriptase and d(pN)6, then amplified by PCR using One Taq 2 Master Mix with standard buffer (New England BioLabs) and a series of primer pairs spanning genomic segments of about 1500 bp. Following purification, the amplified DNAs were then subjected to direct sequencing as described previously^24^.

### Immunoblot analysis

Huh-7.5 cells infected with intergenotyic core viruses at 10 TCID50/cell were lyzed at 72 h, 96 h, or 120 h post-infection in NuPAGE LDS sample buffer (Life Technologies) and HEK293 cells transfected with core expressing DNAs were lyzed at 48 h post-transfection in a CHAPS buffer (Tris 50 mM pH7.5, NaCl 140 mM, EDTA 5 mM, Glycerol 5%, CHAPS 1%), respectively, containing 0.71 M 2-mercaptoethanol, protease inhibitor (Roche) and phosphatase inhibitor (Pierce) cocktails. Protein extracts were incubated for 10 min at 95 °C (NuPAGE LDS extracts) or at 65 °C (CHAPS extracts), loaded onto NuPAGE 4–12% Bis-Tris gels (Life Technologies), separated by sodium dodecyl sulfate-polyacrylamide gel electrophoresis (SDS-PAGE) in NuPAGE MES SDS running buffer (Life Technologies), and transferred onto polyvinylidene difluoride (PVDF) or nitrocellulose membranes (GE Healthcare Life Sciences). Membranes were saturated in PBS containing 0.1% Tween-20 (PBS-T) and 5% dry skimmed milk at room temperature for 1 h and incubated overnight at 4 °C with primary antibodies diluted in PBS-T containing 1% dry skimmed milk. Primary antibodies used are listed in Supplementary Table 3. Following incubation with appropriate secondary antibodies conjugated to DyLight 800 or 680 (Thermo Scientific), proteins were visualized by using the Odyssey CLx imaging system (Li-Cor Biosciences, Lincoln, Nebraska. USA).

### Immunofluorescence and image analysis

Huh-7.5 cells grown on glass coverslips were infected at 10 TCID50/cell and fixed at 5 days post-infection using 4% paraformaldehyde. Cells were permeabilized with 0.2% Triton X-100 and incubated for 15 min with 5% donkey and fetal calf sera and then for 1 h at room temperature with antibodies specific for HCV core or β-catenin (as listed in Supplementary Table 3) diluted in PBS containing 1% donkey and fetal calf sera. Bound primary antibodies were revealed with 488 or 647 Alexa Fluor conjugated antibodies, as indicated in Supplementary Table 3. Nuclei were labeled with 4-6-diamidino-2-phenylindole (DAPI) (Life Technologies) and coverslips were mounted in ProLong Diamond Antifade Mountant (Thermo Fisher). Images of 23 individual cells per condition were acquired with a confocal inverted LSM700 microscope (Zeiss) using a 63X oil immersion objective. Sequential frame averaged scans were set up for each fluorophore to eliminate emission crosstalk. In infected cultures, only effectively infected cells were considered on the basis of HCV core immunolabeling. All Z-stack images were saved in a 16-bit TIFF format with a resolution of 512 × 512 pixels. 3D images were deconvolved with Huygens Essential software (Version X11, Scientific Volume Imaging BV, Hilversum, The Netherlands). Deconvolved images centered on single cells were subjected to object segmentation using Object Analyzer. Object analysis (core, β-catenin and DAPI channels taken individually) and colocalization analyses of intersecting volumes between two channels of interest (DAPI and β-catenin) were performed using Huygens Professional software (Version X11). For each channel, an individual threshold was selected and maintained for all processed samples. Voxels with intensity above the established threshold were quantified and the number of intersecting voxels between channels of interest was calculated for each individual cell analyzed. Voxels may then be translated in volumes expressed in µm^3^. Intersecting volumes of β-catenin and DAPI channels provide a quantification of nuclear β-catenin. Microsoft Excel and GraphPad Prism 8 softwares were used for data analysis, chart generation and statistical analyses.

### Statistical analyses

Student’s tests were used to compare the effects of various core sequences in core-expressing DNA transfected or HCV infected cells. The normal distribution of data was assessed in GraphPad Prism software. Differences were considered to be significant when P values were <0.05. The Holm-Sidak method (GraphPad Prism) was used to evaluate the statistical significance of the intersecting volume analyses in immunofluorescence experiments.

### Data availability statement

All data generated or analysed during this study are included in this article (and its Supplementary Information files).

### Accession codes

Genome-length cDNA of the prototypic H77 strain of HCV subtype 1a (1aH77)^48^: GenBank accession #AF009606; Genome-length cDNA of JFH1 strain of HCV subtype 2a^23^: GenBank accession #AB047639; HCV core sequences reported in the present study (1aC, 1aCvar, 4aR, 4fC): European Nucleotide Archive (ENA) #ERZ664228, ERZ655053, ERZ655054 and ERZ672786.

## Electronic supplementary material

Supplementary information
