# Supplementary material for: Differential regulation of the Wnt/β-catenin pathway by hepatitis C virus recombinants expressing core from various genotypes
Source: Sci Rep. 2018 Jul 25;8:11185. doi: 10.1038/s41598-018-29078-2 (PMC6060129; doi:10.1038/s41598-018-29078-2)
Supplement: Supplementary file 1 — Supplementary information [file 41598_2018_29078_MOESM1_ESM.pdf]

Differential regulation of the Wnt/β-catenin pathway by hepatitis C virus recombinants expressing core from various genotypes

Stephanie Aicher, Athanasios Kakkanas, Lisette Cohen, Brigitte Blumen, Gabriela Oprisan, Richard Njouom, Eliane F. Meurs, Penelope Mavromara, and Annette Martin

Supplementary Figure 1. Co-localization of core from various genotypes and lipid droplets in infected cells. Huh-7.5 cells were infected with the indicated viruses at a multiplicity of infection of 10 TCID50 / cell or mock-infected (noninfected) and fixed at 5 days post-infection. Cells were then labeled for nucleus (DAPI, blue), adipose differentiation-related protein (ADRP, red) as a surface marker for lipid droplets, and HCV core (green). Deconvolved images of DAPI and ADRP labeling (left), core labeling (middle), and merged signals (right) are shown.

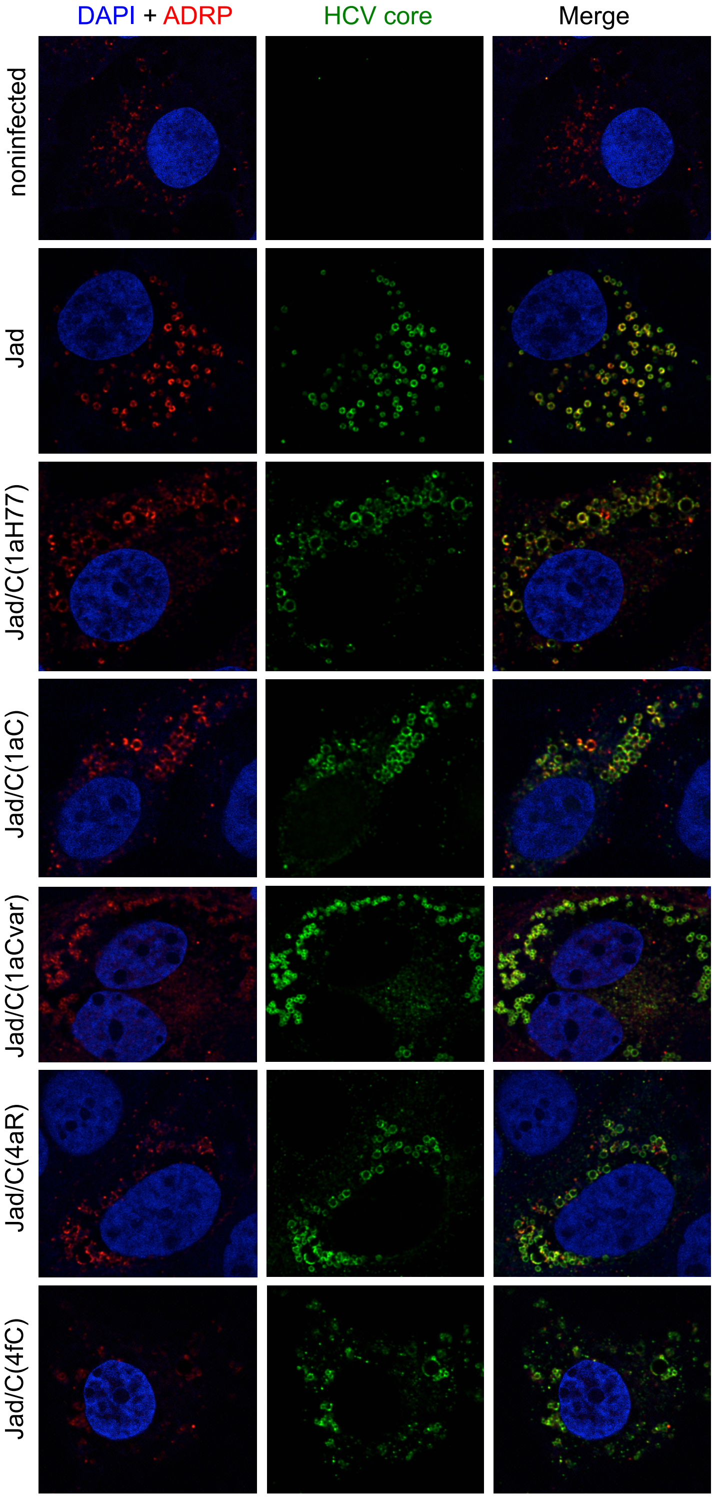

Supplementary Figure 2. Detection of immuno-reactive core polypeptides using polyclonal antibodies raised against HCV core amino acids 1-120. Protein extracts were prepared from cells transfected in the absence of RNA (mock), with the indicated genome-length reporter recombinant RNAs [Jad-2EIL3/C(XX)], or with controls including Jad-2EIL3 (parent) RNA, replication-deficient Jad-2EIL3/GAA or assembly-deficient Jad-2EIL3/ΔEp7 RNAs. Proteins were separated by SDS-PAGE and probed with polyclonal antibodies raised against amino-acids 1-120 of HCV core.

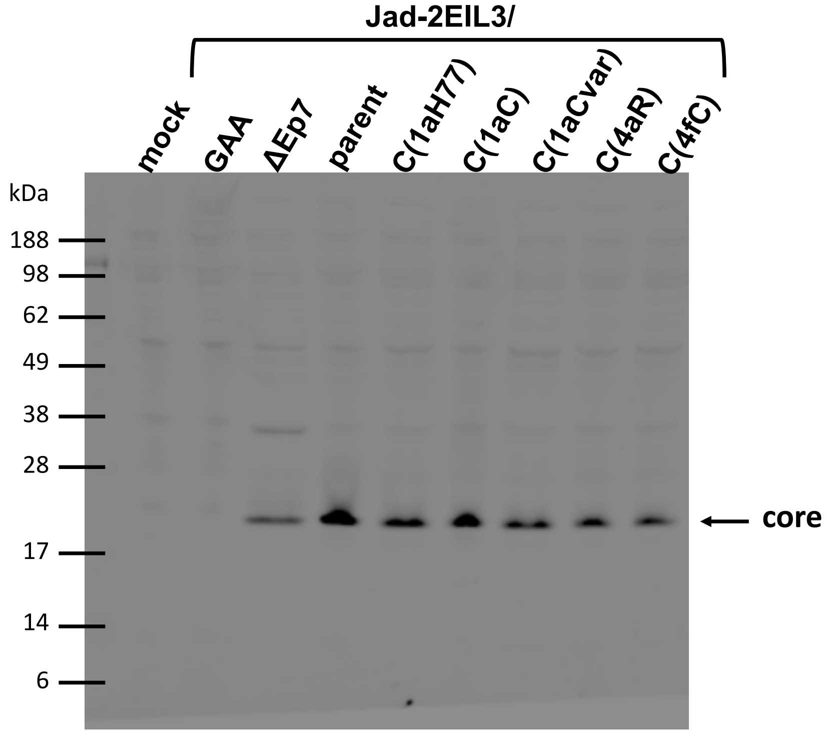

Supplementary Figure 3. Detection of ß-catenin in 3D images centered on cell nuclei. A representative set of 3D-rendered deconvolved images centered on cell nuclei that were used for object segmentation is shown for cells infected with each of the indicated recombinant virus and for noninfected cells. Cell nuclei were labeled with 4-6-diamidino-2-phenylindole (DAPI, blue) and with anti-β-catenin monoclonal antibodies (yellow). Only infected cells were considered for the recombinant viruses, as confirmed by core immuno-labeling (not shown here for clarity).

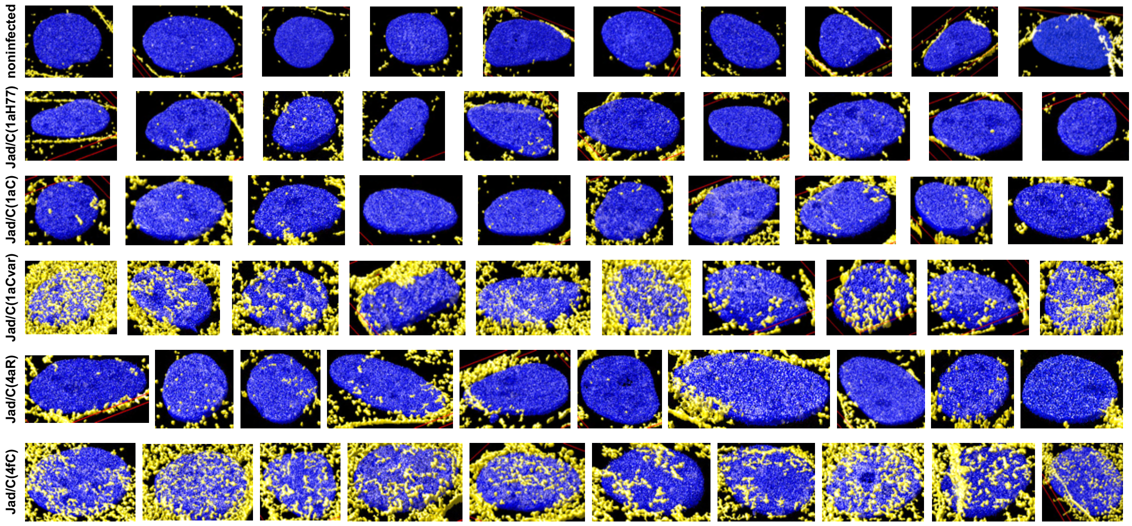

Supplementary Figure 4. Quantification of total β-catenin abundance in cells. Huh-7.5 cells infected with the indicated viruses or noninfected cells (NI) were labeled for nucleus, core and β-catenin, as shown in Fig. 6a. Deconvolved Z-stacked images were subjected to object analysis using Huygens Professional software and total volumes of β-catenin (present at the cell surface, within the cytosol and the nucleus) were quantified per cell for a total of 23 cells per condition. Statistical analyses with respect to values obtained in noninfected cells are indicated above each group of virus-infected cells (in grey characters), while statistical analyses between two related variants are indicated in black characters above brackets. These statistical analyses were performed according to the Holm-Sidak method and are coded as follows: $P<0.05$ (*), $P<0.005$ (**), $P<0.001$ (***).

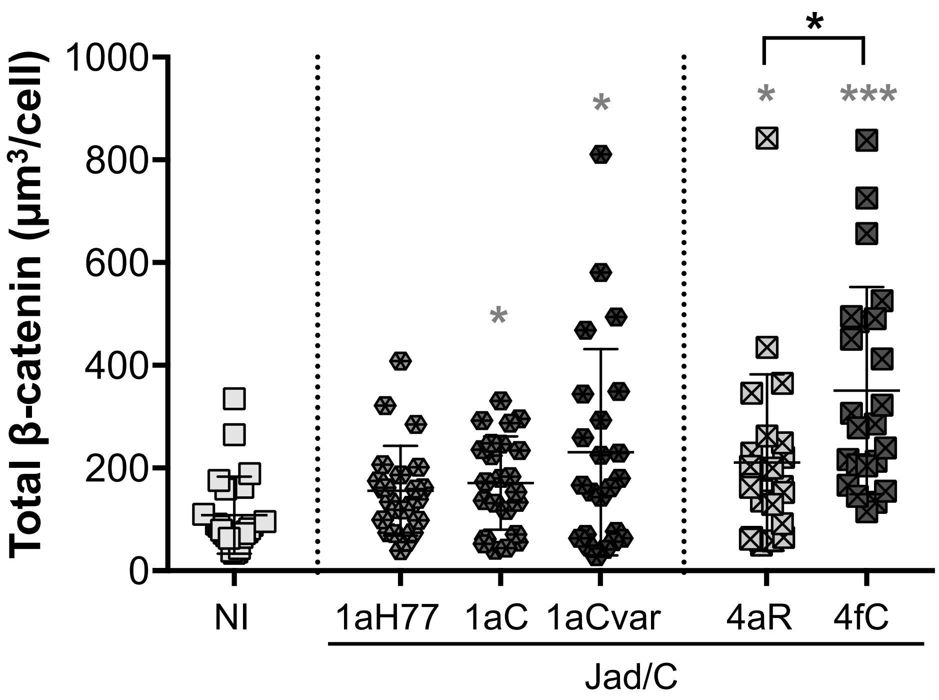

Supplementary Figure 5. Full-length immunoblot from which Fig. 1b images were extracted. Protein extracts prepared from cells transfected with pCI DNA (empty vector) or pCI expressing the indicated core proteins were electrophoresed and transferred onto membranes. Blots were probed with a mixture of 4 monoclonal antibodies including anti-actin and anti-HCV core antibodies.

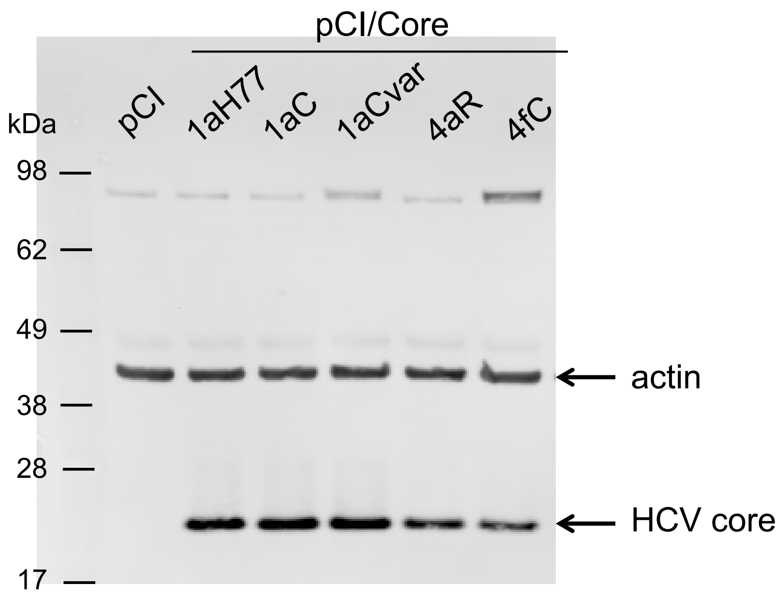

Supplementary Figure 6. Full-length immunoblots from which Fig. 4d images were extracted. Protein extracts were prepared at the indicated days post-infection (d.p.i.) from noninfected cells (Non infected or NI) or from cells infected with the indicated viruses, electrophoresed and transferred onto membranes. Blots were probed with a mixture of 4 monoclonal antibodies including anti-actin and anti-HCV core antibodies (left images), then the entire or top-half membranes were stripped and re-probed with anti-HCV NS5A antibodies (right images).

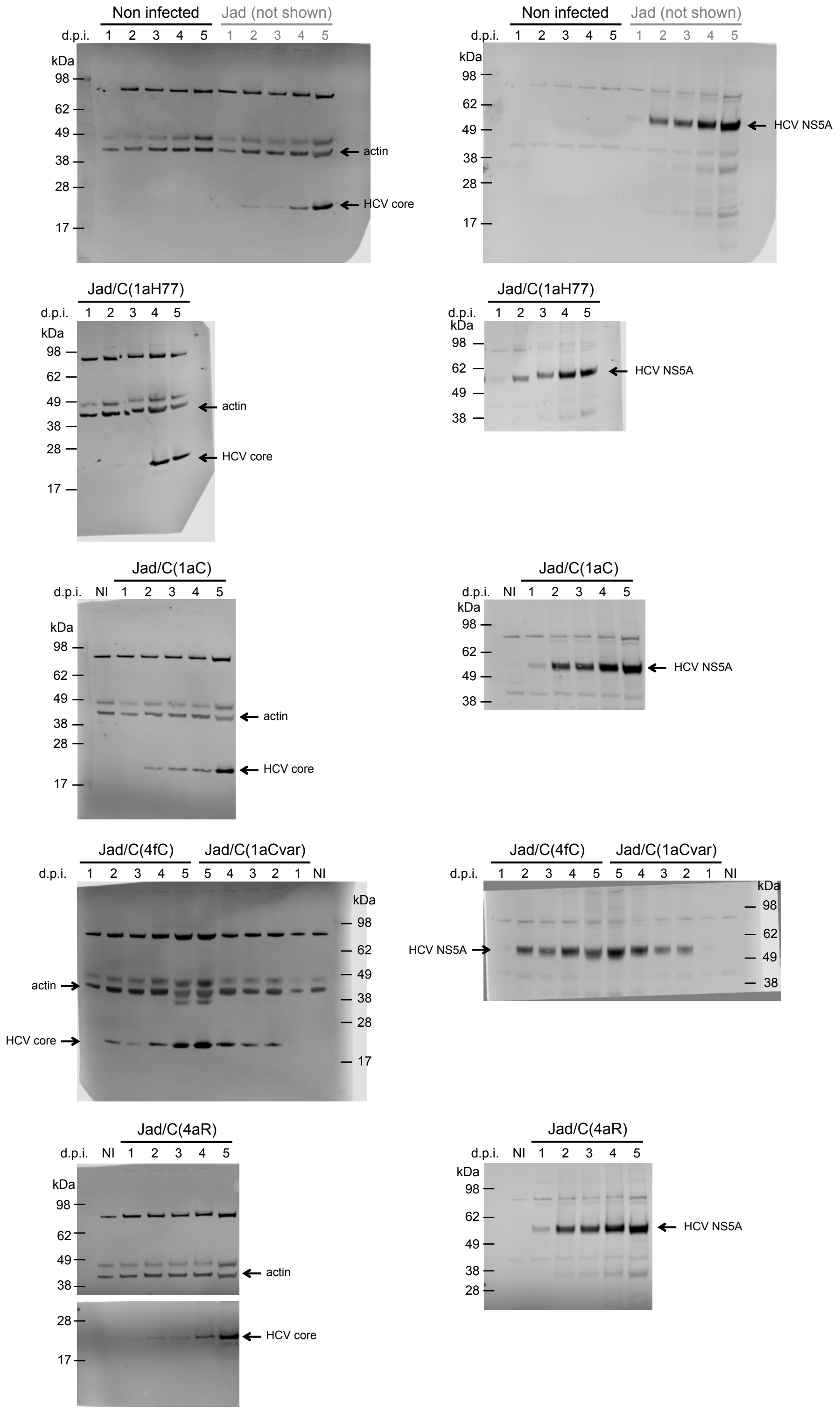

Supplementary Table 1. Primer pairs used to amplify the core coding sequences of the indicated viral strains [a]

Primer name	Primer sequence (listed 5’ to 3')
4aR/4fCF	CTAGCTAGCATGAGCACGAATCCTAA
4aRR	GCTCTAGATCAGGTCGAAGCGGGGA
4fCR	GCTCTAGATCAAGCCGAGGCGGGAA

[a]F: forward, R: reverse

Supplementary Table 2. Primer pairs used to reverse transcribe and amplify the indicated human mRNA sequences [a]

Primer name	Primer sequence (listed 5' to 3')
**c-*MYC*F**	CACCAGCAGCGACTCTGA
**c-*MYC*R**	GATCCAGACTCTGACCTTTTGC
***CyclinD1*F**	CCGTCCATGCGGAAGATC
***CyclinD1*R**	GAAGACCTCCTCCTCGCACT
***TBX3*F**	GCAGCTTTCAACTGCTTCG
***TBX3*R**	CCTCGCTGGGACATAAATCT
***BC002942*[b]F**	GTGGCCACCTTCCTAATTGA
***BC002942*[b]R**	GTTGTAGTTGCCGGTGATGA
***HMBS*[c]*164*F**	TGGCAATGCGGCTGCA
***HMBS*[c]*164*R**	TGGGTACCCACGCGAATCAC
***SFRS4*[d]F**	AAAAGTCGGAGCAGGAGTCA
***SFRS4*[d]R**	CTCTTCCTGCCCTTCCTCTT
***GAPDH*[e]F**	AGCCACATCGCTCAGACAC
***GAPDH*[e]R**	GCCCAATACGACCAAATCC
***AXIN2*F**	GAGTAGCGCTATGTTGGTGACT
***AXIN2*R**	GGCTGACACGGTGGGGTCTC
***FNDC3B*F**	GAATGGAGATGCAGCTCAGCAG
***FNDC3B*R**	GAATGGGAGGAATGGATCCATTGA
FASNF	CTTCCGAGATTCCATCCTACGC
FASNR	TGGCAGTCAGGCTCACAAACG

[a]F: forward, R: reverse; [b,c,d,e] houskeeping genes: [b]BC002942, lipase maturation factor 2; [c]HMBS, hydroxymethyl-bilane synthase; [d]SFRS4, serine and arginine rich splicing factor 4; [e]GAPDH, glyceraldehyde-3-phosphate dehydrogenase

Supplementary Table 3. Commercially-available antibodies used in this study[a]

Antibody	Company	Catalog number	Working concentration (μg/mL)
Rabbit anti-β-catenin monoclonal antibody (E247)	Abcam (Cambridge, UK)	ab32572	0.8
Mouse anti-β-actin monoclonal antibody (AC-15)	Abcam (Cambridge, UK)	ab6276	0.5
Mouse anti-HCV core monoclonal antibody (1851)	Santa Cruz (Santa Cruz, CA, USA)	sc-58144	WB 0.2<br>IF 0.1
Mouse anti-HCV NS5A monoclonal antibody	Biofront (Tallahassee, FL, USA)	HCV-7B5	1
Mouse anti-HCV NS3 monoclonal antibody	Biofront (Tallahassee, FL, USA)	HCV-2E3	0.3
Goat anti-mouse IgG (H&L), DyLight 800 conjugated	Thermo FisherScientific (Waltham, USA)	SA5-35521	0.05
Goat anti-rabbit IgG H&L, Alexa Fluor 647 conjugated	Abcam (Cambridge, UK)	ab150079	4
Goat anti-mouse IgG H&L, Alexa Fluor 488 conjugated	Thermo FisherScientific (Waltham, USA)	A11029	4

[a]WB: western blot, IF: immunofluorescence
